# Ventricular Tract Hemorrhage Following Intracranial Nail Removal: Utility of Real-time Endovascular Assistance

**DOI:** 10.3389/fneur.2016.00112

**Published:** 2016-07-14

**Authors:** Robert C. Rennert, Jeffrey A. Steinberg, Jayson Sack, J. Scott Pannell, Alexander A. Khalessi

**Affiliations:** ^1^Department of Neurosurgery, University of California San Diego, San Diego, CA, USA

**Keywords:** nail gun, penetrating brain trauma, cerebral embolization

## Abstract

Penetrating brain trauma commonly results in occult neurovascular injury. Detailed cerebrovascular imaging can evaluate the relationship of intracranial foreign bodies to major vascular structures, assess for traumatic pseudoaneurysms, and ensure hemostasis during surgical removal. We report a case of a self-inflicted intracranial nail gun injury causing a communicating ventricular tract hemorrhage upon removal, as well as a delayed pseudoaneurysm. Pre- and post-operative vascular imaging, as well as intra-operative endovascular assistance, was critical to successful foreign body removal in this patient. This report demonstrates the utility of endovascular techniques for the assessment and treatment of occult cerebrovascular injuries from intracranial foreign bodies.

Penetrating brain trauma is less common than closed head injury, with outcomes dependent on the penetrating object (1, 2). Gunshot wounds to the head comprise the majority of penetrating head traumas and are the most lethal due to their ballistic properties (1, 3). Accidental or intentional penetrating brain injuries from nail guns are a well-described phenomenon (4–8). Although nail guns are capable of firing projectiles at high speeds (up to 427 m/s), intracranial nails tend to act more like low-velocity missiles and cause only limited local injury (4). Aside from infection, a major concern with nail gun and other low-velocity penetrating head trauma is vascular injury. These situations necessitate detailed cerebrovascular imaging to evaluate the relationship of the missile to major vascular structures and to assess for traumatic pseudoaneurysms.

We report here clinical and neuroimaging findings from a patient with an intracranial nail gun injury, a communicating ventricular tract hemorrhage upon nail removal, and a delayed pseudoaneurysm, whose treatment was augmented with neuroendovascular techniques.

## Case Report

A 62-year-old male with a history of depression was found several hours after a self-inflicted nail gun injury to the left temple. He was brought to an area hospital awake but exhibiting severe expressive aphasia. He was sedated, intubated, and transferred to our facility for further management.

**Figure 1:**
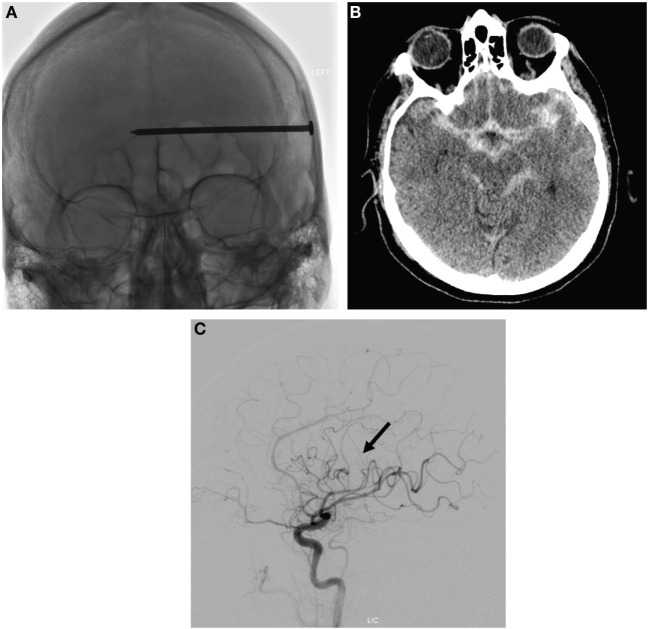
**Assessment of initial injury from intracranial nail**. **(A)** AP skull plain radiograph demonstrating nail penetration depth. **(B)** Axial image from head CT demonstrating basilar traumatic subarachnoid hemorrhage. **(C)** Lateral projection of a left internal carotid angiogram demonstrating nail positioning in the vicinity of a left M3 branch (nail head highlighted by black arrow), with no aneurysms, vessel occlusions/transections, or active extravasation.

Broad-spectrum antibiotics and anti-seizure medications were started. Head CT demonstrated a large nail horizontally traversing the left cerebral hemisphere, with its tip terminating near the body of the right lateral ventricle. Subarachnoid hemorrhage was seen in the bilateral frontoparietal sulci, sylvian fissures, and the basilar and prepontine cisterns, with a small amount of intraventricular hemorrhage in the occipital horns of the lateral ventricles. Vascular imaging demonstrated a nail trajectory immediately subjacent to a left M3 branch, with no aneurysms, pseudoaneurysms, vessel occlusions/transections, or active extravasation (Figure 1).

Nail removal was planned in the angiography suite with endovascular assistance for rapid hemostasis if needed. Following baseline angiograms of the left anterior circulation, a microcatheter and guidewire were positioned in the petrous segment of the left internal carotid artery for rapid selective embolization.

**Figure 2:**
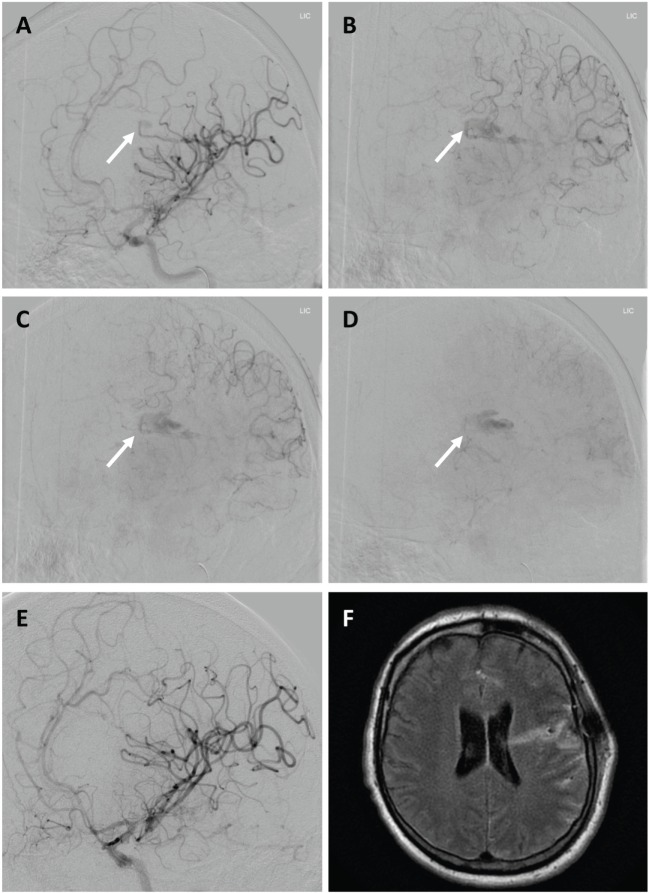
**Ventricular tract hemorrhage following intracranial nail removal**. **(A–D)** Serial oblique projections of a left internal carotid angiogram immediately after nail removal, demonstrating active hemorrhage (white arrows) from a small left M3 branch extending down the puncture tract into the lateral ventricle. **(E)** Lateral left internal carotid angiogram confirming resolution of hemorrhage after no active extravasation was seen on serial M3 microcatheterizations. No aneurysmal dilatation or flow-limiting stenosis is seen. **(F)** Axial T2-weighted flair brain MRI demonstrating only mild local inflammation 5 days after nail removal.

The nail head was then identified following a small skin incision and superficial dissection and removed from the cranium using a double-action Leksell rongeur. An angiogram was subsequently obtained. Immediate injection of the left internal carotid revealed active contrast extravasation from a small M3 branch arising from the inferior trunk in the area of the anterior parietal artery and extending down the puncture tract into the left lateral ventricle (Figures 2A–D). Surgiflo (prepared as per manufacturer instructions; Ethicon, Somerville, NJ, USA) was injected into the lateral-most portion of the puncture wound tract, extending through the left parietal lobe for bridging hemostasis and attention was turned to a definitive endovascular treatment. The left MCA trunks were then serially micro-catheterized in preparation for embolization, but each demonstrated no active extravasation. Resolution of hemorrhage was confirmed on repeat internal carotid angiogram (Figure 2E). Post-procedure head CT demonstrated a small left lateral ventricle IVH, for which an external ventricular drain (EVD) was placed. This was converted to a ventriculoperitoneal shunt following an unsuccessful EVD wean approximately 3 weeks after nail removal. Post-operative MRI revealed gliosis along the nail tract, but not global infectious or ischemic processes (Figure 2F).

**Figure 3:**
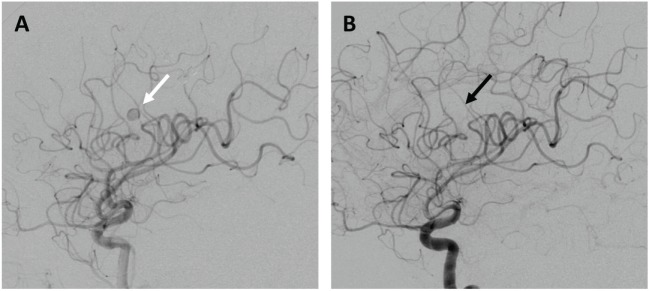
**Delayed pseudoaneurysm formation**. **(A)** Left internal carotid angiogram demonstrating a left anterior parietal MCA branch pseudoaneurysm (white arrow), successfully treated *via* proximal branch embolization [**(B)**; black arrow highlighting area of branch sacrifice].

At 1 month follow-up, the patient experienced only short-term memory problems and mild proximal LLE weakness, which resolved by 3 months. A delayed pseudoaneurysm in the anterior parietal branch of the left MCA was identified on 3-month follow-up imaging, and uneventfully treated *via* branch sacrifice with *n*-butyl cyanoacrylate (*n*-BCA) liquid embolic glue (Figure 3). Delayed seizures were medically managed. At 1 year follow-up, only mild short-term memory deficits persisted.

## Discussion

This report demonstrates how intracranial nails (or other similar low-velocity penetrating objects) can be safely extracted with careful surgical planning and post-operative follow-up to assess for and treat cerebrovascular injury.

The decision to remove intracranial foreign bodies is multifactorial. Conservative management is typically pursued with objects that are deeply situated or in eloquent tissue, are irregular and would cause significant injury with extraction, or are high velocity (i.e., bullets, shrapnel) and thereby cause more extensive injuries and theoretic (although unproven) bactericidal heating upon entry (1, 9). Conversely, superficial, lower velocity objects with a regular shape, such as nails, are good candidates for removal to mitigate infectious and corrosive risks.

Infections following intracranial penetrating trauma of all types are nonetheless on the order of 2–5% for bullet or shrapnel wounds, and slightly higher for traumas from other causes (10). Causative organisms for superficial infections are most commonly Gram-positive bacteria, such as coagulase-negative staphylococci, while deeper infections are more likely *Staphylococcus aureus*, Gram-negative facultatively aerobic bacteria, or mixed. A course of prophylactic broad-spectrum antibiotics is thus recommended following penetrating intracranial trauma (10) and was administered in this case.

Cerebrovascular injuries at the time of injury and during foreign body removal can be life-threatening. In this case, the patient had no obvious pre-operative vascular insults. The nail was nonetheless removed on a biplane fluoroscopic table with a microcatheter positioned for rapid embolization in the event of hemorrhage from an occult vascular injury. The hemorrhage that occurred would not have been otherwise visible, or self-tamponaded given the communication of the nail tract with the lateral ventricle. While the injection of Surgiflo down the nail tract led to hemostasis before embolization was required, the utility of rapid endovascular embolization in this setting is clear. Surgical treatment, while previously described for removal of intracranial nails impacting the cerebrovasculature (11), would have required significant dissection in highly eloquent territory to secure the deep arterial source. Instead, Surgiflo and collapse of the tract lead to adequate hemostasis and immediate angiography confirmed a successful result and positioned the patient for a more definitive endovascular treatment in the event of refractory extravasation. While other reports describe nail removal following endovascular arterial embolization (12, 13), this is to our knowledge the first report of real-time microangiography for monitoring and potential control of a perioperative hemorrhage.

Immediate or delayed pseudoaneurysm development is also described following traumatic or iatrogenic cerebral artery injury and can be successfully managed with endovascular techniques (4, 14–16). Delayed pseudoaneurysms can occur years after the initial injury, but on average will be seen within 2–3 weeks (16). Serial vascular imaging is thus required in these patients, and in this case identified and allowed for the uneventful treatment of a life-threatening delayed pseudoaneurysm.

In conclusion, this case highlights the use of endovascular approaches to help identify and treat occult vascular injuries associated with intracranial foreign bodies.

## Ethics Statement

Written consent was obtained for all clinical procedures as per University of California protocol.

## Author Contributions

RR, JAS, JS, JP, and AK contributed to the clinical care of this patient, as well as the drafting and editing of the manuscript.

## Conflict of Interest Statement

The authors have no disclosures concerning the materials or methods used in this study or the findings specified in this paper. The reviewer NM and handling Editor declared their shared affiliation, and the handling editor states that the process nevertheless met the standards of a fair and objective review.
